# Prosthetic management of an ocular defect utilizing a novel threaded iris fabrication technique

**DOI:** 10.1002/ccr3.7287

**Published:** 2023-06-01

**Authors:** James Dudley, Jane Pellew, Nafij Bin Jamayet

**Affiliations:** ^1^ Adelaide Dental School The University of Adelaide South Australia Adelaide Australia; ^2^ School of Dentistry International Medical University Kuala Lumpur Malaysia

**Keywords:** maxillo‐facial prosthetics, multi‐disciplinary, ocular prosthesis, threaded iris technique

## Abstract

The conservative prosthodontic construction of an ocular prosthesis utilizing our novel threaded iris fabrication technique required high time and prosthodontic resource inputs and produced a lifelike aesthetic result.

**Abstract:**

Patients with ocular defects frequently present with significant local anatomical deficiencies and complex histories and require extensive time and resource inputs to treat. This case report describes the conservative management of an ocular defect completed in a postgraduate prosthodontics clinical residency program utilizing a novel threaded iris fabrication technique.

## Graphical abstract

Initial presentation of the aquired eye defect with inflamed tissue and a contracted socket with superior sulcus deformity.

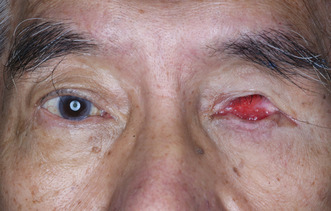

## 1 INTRODUCTION

Maxillo‐facial prosthetics is the highly specialized division of prosthodontics focused on the restoration and/or replacement of stomatognathic and craniofacial structures with prostheses that may or may not be removed on a regular or elective basis.^1^ Maxillo‐facial prosthetic treatment is typically delivered using a multi‐disciplinary and/or a team approach and performed by prosthodontists in medical institutions. However, general dentists can provide invaluable assistance complementing the routine maintenance of such patients through an understanding of referral processes and identifying and coordinating prosthesis maintenance and repair procedures.^2^

A maxillo‐facial prosthesis aims to restore elements of mastication, deglutition, normal speech and aesthetics,^3^, ^4^ and patients with maxillo‐facial defects clearly benefit from treatment. In accomplishing treatment objectives, the patient's return to society is expediated. Frequently, the uniqueness of the maxillo‐facial treatment process is demonstrated in combining elements of art and science into the surgical, prosthetic or combined surgical/prosthetic phases.

The loss of an eye results is a traumatic event in a patient's life and results in facial disfigurement, functional limitations and negative psychological impact.^5^ Orbital defects may result from surgical ablation of benign or malignant tumors, congenital malformations or traumatic injury.^6^ After orbital enucleation, the residual musculature has a pronounced influence on the possible prosthesis types.^7^

The types of prosthetic rehabilitation following surgical removal of the eye are broadly divided into ocular and orbital prostheses.^8^ An ocular prosthesis comprises an acrylic shell that cosmetically restores tissue loss, whereas an orbital prosthesis replaces additional structures such as eyelids, eyelashes and eyebrows.^9^ Prostheses for ocular and orbital defects are constructed from materials including polymethyl methacrylate, polyurethane elastomer, silicone elastomer and urethane backed medical grade silicone.^10^ The prostheses are retained by adhesives, mechanically using anatomical undercuts, spectacle frames or osseointegrated extra‐oral implants.^10^ The option to construct an ocular prosthesis represents a viable and economical alternative when the aesthetic demands are beyond the surgical reconstructive capacity.

There is minimal teaching of maxillo‐facial prosthodontics in undergraduate Australian dental curricula, however it is a required competency in postgraduate training programs.^11^ This likely relates to the high level of specialization, case complexity, required skills and experience, frequency of cases seen in practice, and the required underlying level of procedural knowledge. The following ocular prosthesis case incorporating the completion of all clinical and laboratory stages was completed in a postgraduate prosthodontics clinical residency program by international collaboration between two universities. The success of the case was illustrated in the attention to detail, artistic creativity and managing realistic treatment outcomes.

## 2 CASE: OCULAR PROSTHESIS

**FIGURE 1:**
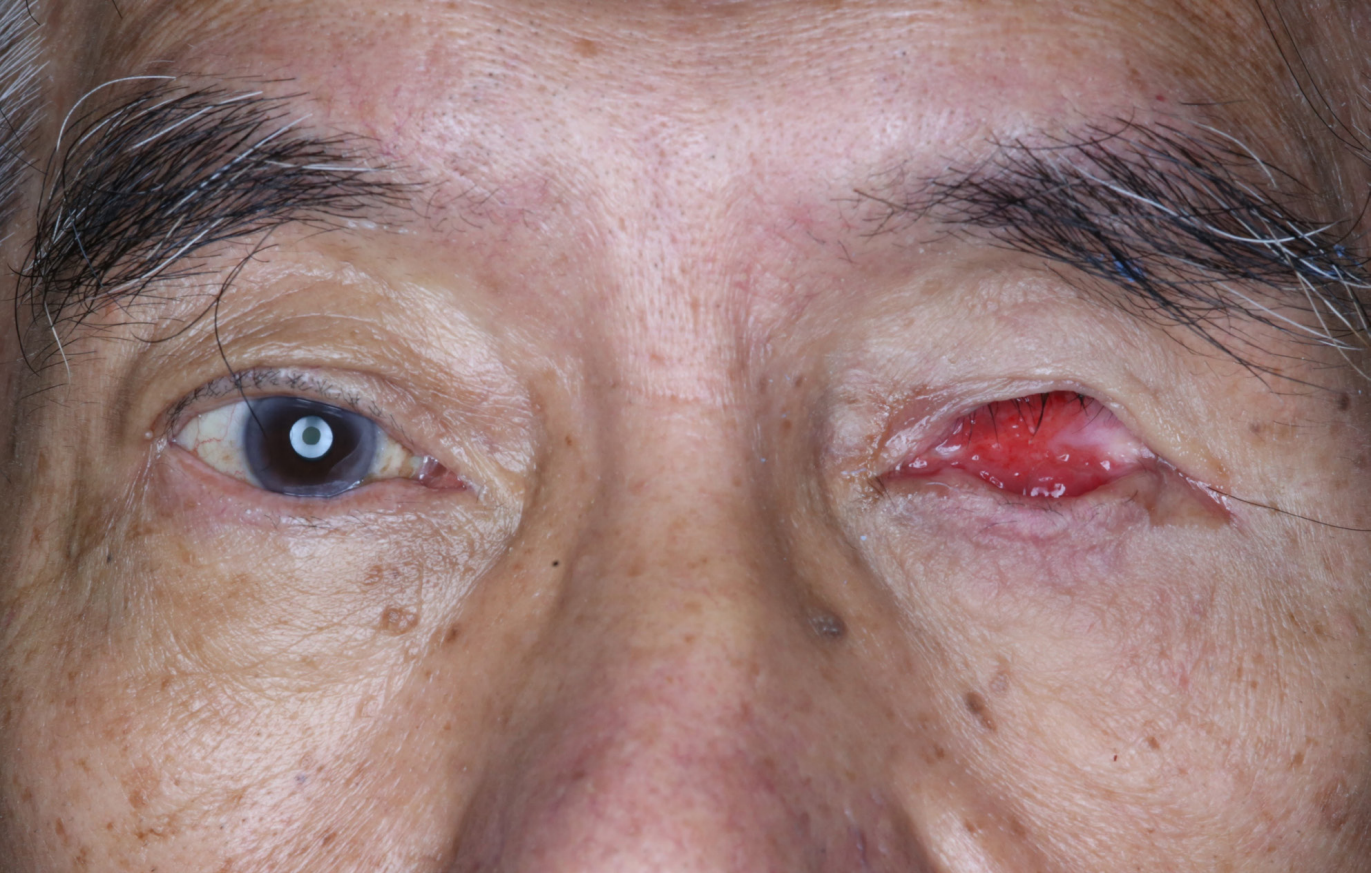
Patient at initial presentation.

A 76‐year‐old male patient presented with a history of discomfort and irritation from his left eye socket (Figure 1). The patient had enucleation of the globe at age 6 due to infection and received several prostheses throughout his childhood. A 14 mm diameter hydroxyapatite ball implant (G‐EYE Modified Hydroxyapatite eye sphere, size MHAE 14, Surgiwear, India) was placed in 2012 and subsequently removed in early 2020 due to exposure with surgical revision performed in late 2020 to increase the socket volume. The existing prosthesis was constructed in early 2022 but had drifted laterally and had an off‐centred iris. Detailed socket examination revealed entropion (the inversion or turning inward of the border of the eyelid against the eyeball) of the upper fornix, deepened superior sulcus with loss of orbital fat, no ptosis, reduced socket volume and an inflamed tissue bed. The diagnosis was of an acquired eye defect with inflamed tissue and a contracted socket with superior sulcus deformity. The available treatment options involved either surgical intervention to further refine the socket or construction of a new prosthesis. Following discussion and obtaining informed consent, the latter treatment option was selected focussed specifically on the provision of a customized ocular prosthesis.

**FIGURE 2:**
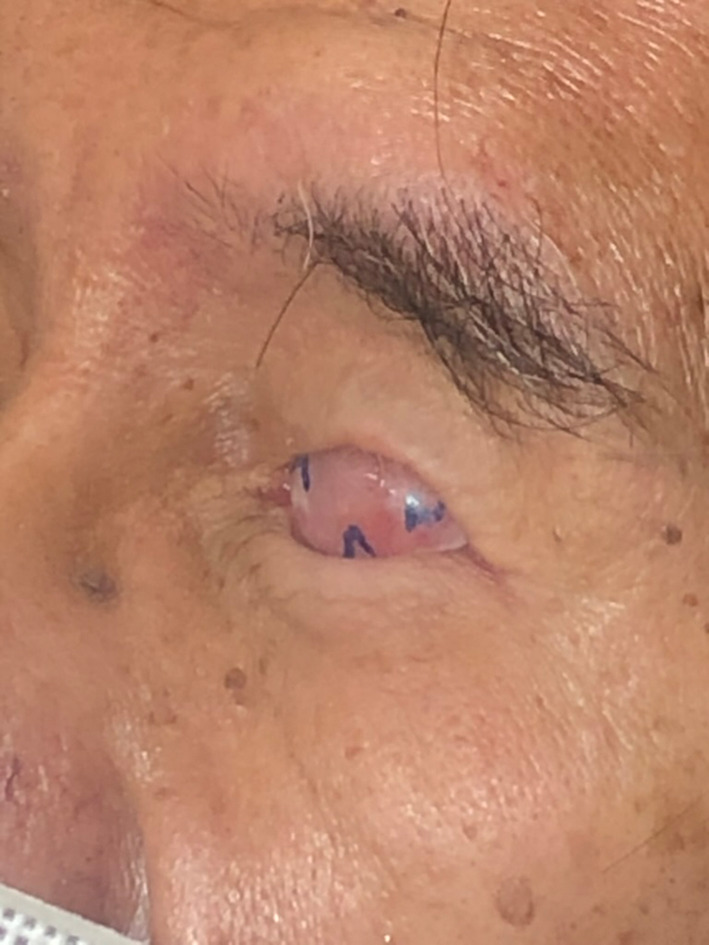
Prefabricated and marked impression tray.

**FIGURE 3:**
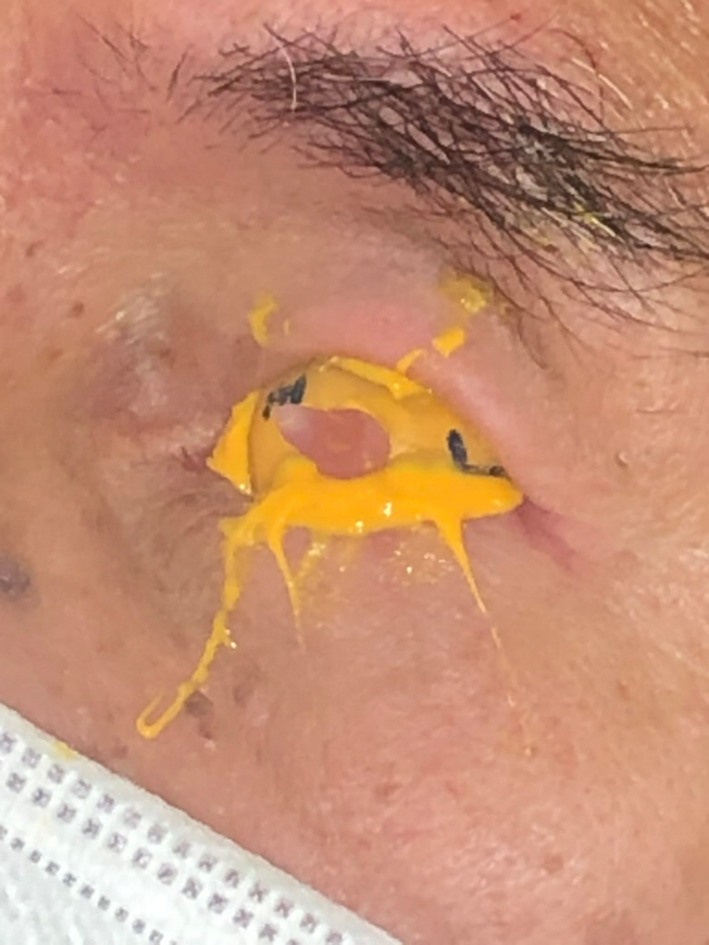
Impression tray with impression.

**FIGURE 4:**
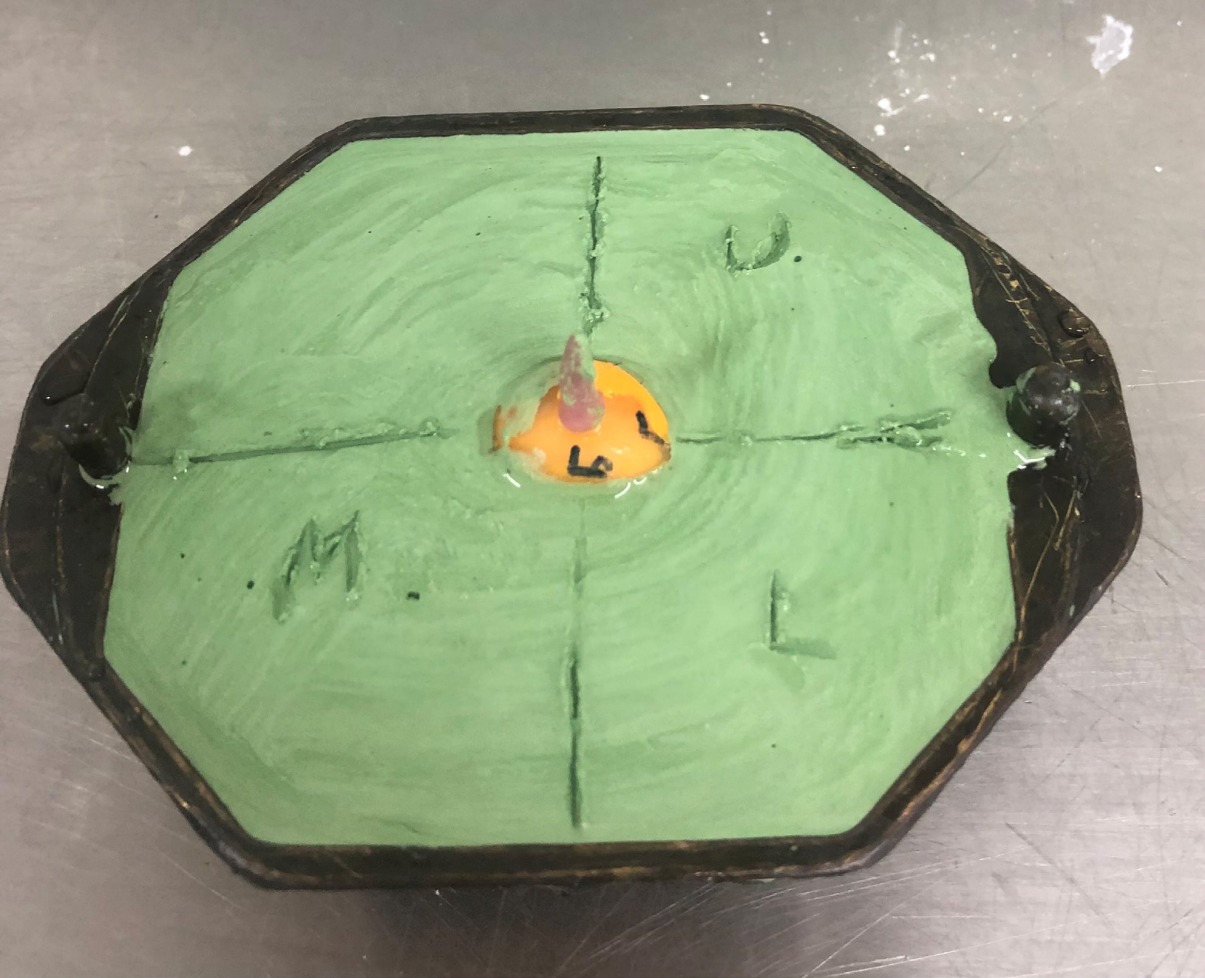
Flasked conformer with resin handle.

The treatment commenced with fitting an appropriately sized prefabricated impression tray selected from our laboratory's non‐proprietary impression tray range, marking the medial and lateral canthus and making an impression with light body polyvinyl siloxane (Aquasil Ultra, Dentsply Sirona, USA) impression material (Figures 2 and 3). The impression was poured in greenstone (Pro‐Solid Green, Saint‐Gobain Formula, France) and flasked to construct a heat cured clear acrylic resin (Vertex Dental, Netherlands) conformer (Figure 4). The conformer was tried in and adjusted with an acrylic bur to ensure appropriate support and fit. The medial and lateral canthus were marked on the conformer, the iris size and position were measured and marked, and the centre of the pupil was marked with wax. The conformer was removed and flasked again in greenstone (Pro‐Solid Green, Saint‐Gobain Formula, France) to transfer the centre of the iris. Heat cured scleral acrylic resin was added (J‐510 Scleral Polymer, Factor II, USA) and cured under 3 MPa for 3 h in a water bath at 100°C to fabricate the sclera prosthesis. The sclera prosthesis was trimmed and polished.

**FIGURE 5:**
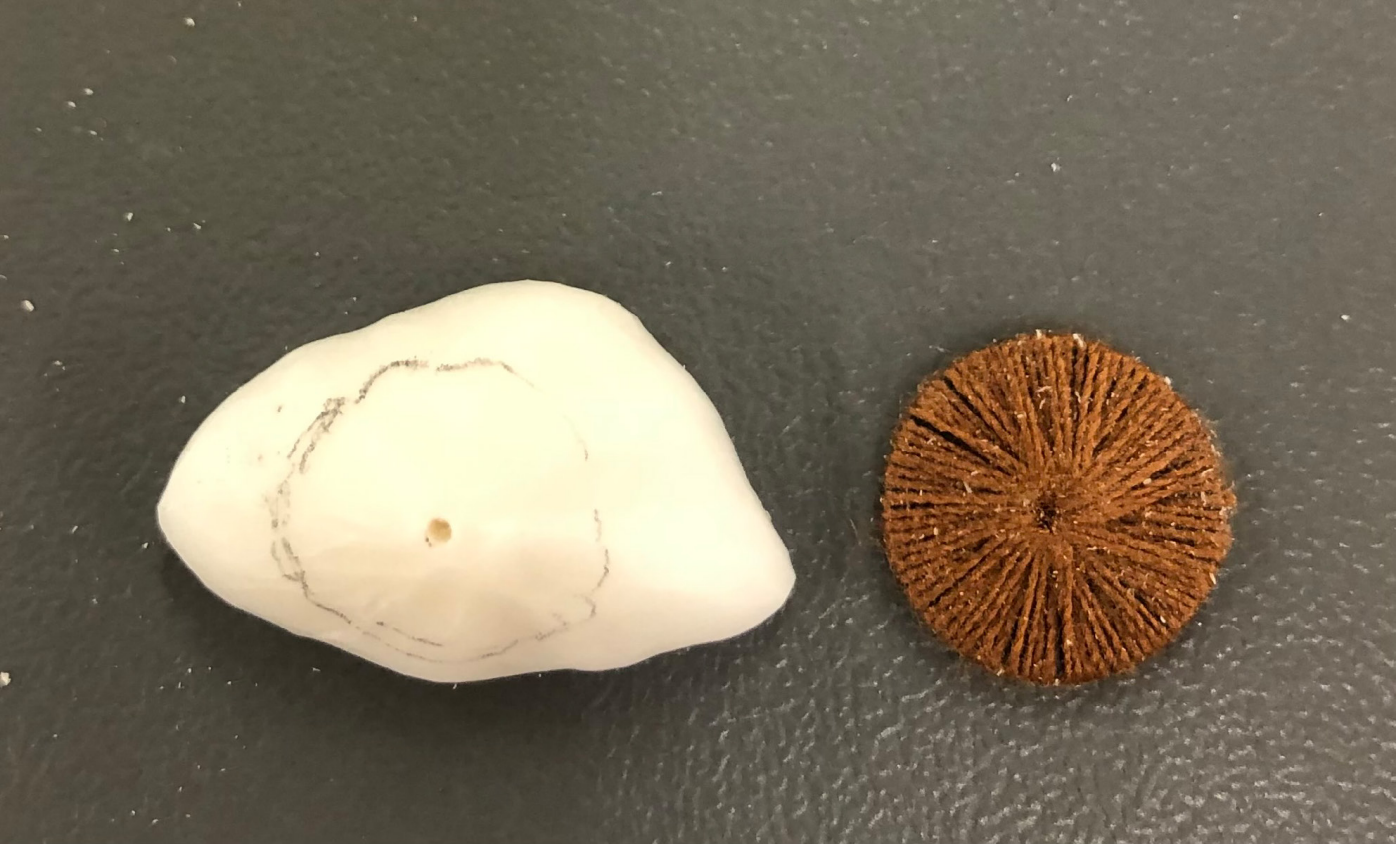
Iris fabrication using the threaded technique.

**FIGURE 6:**
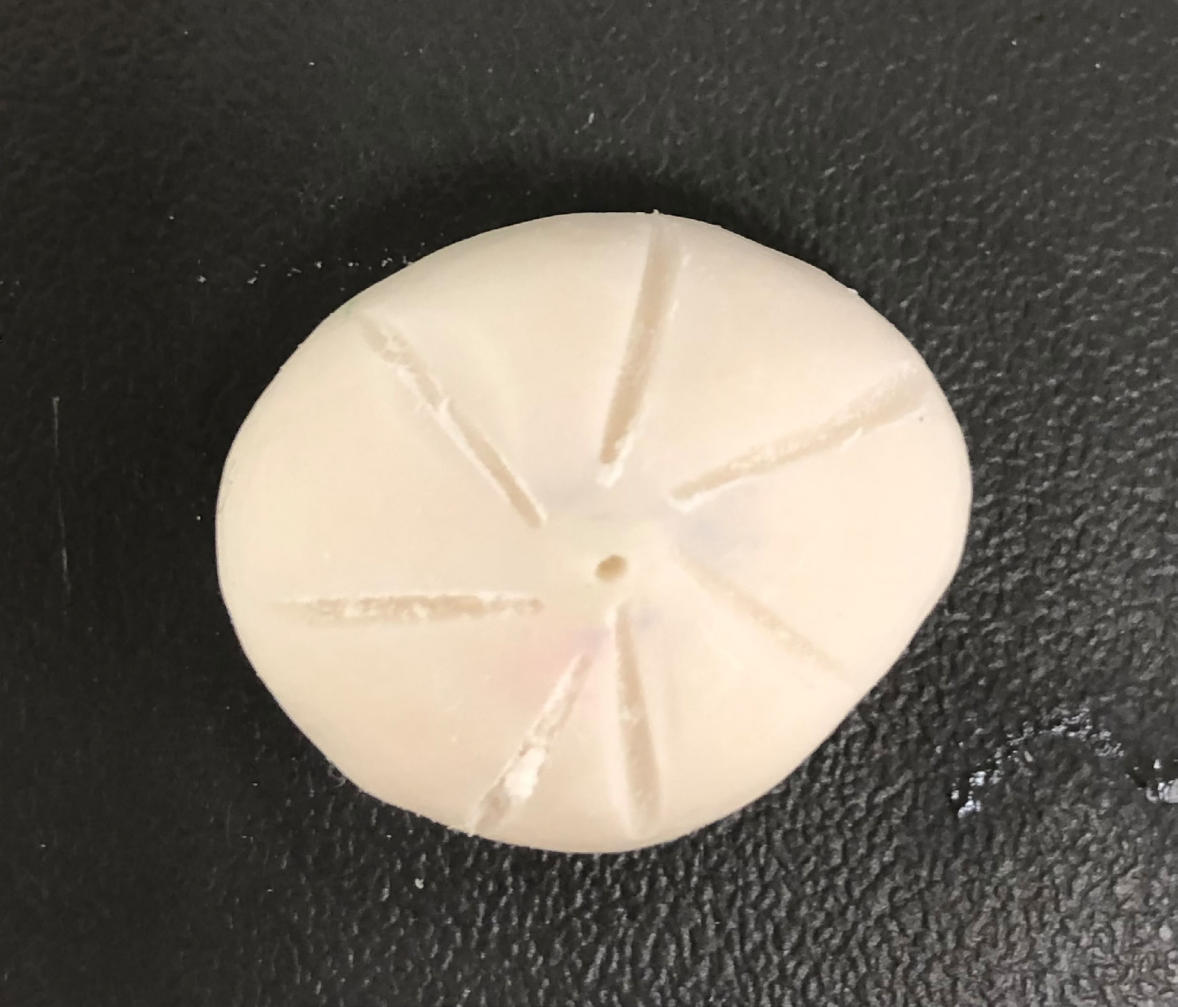
Reducing the sclera prosthesis to facilitate acrylic resin.

The iris was fabricated using the novel threaded technique in preference to the painting technique. The correct iris size was firstly measured in reference to photographs, and a radiograph film trimmed to form the iris wheel. A central hole was created in the iris wheel (radiograph film) and a sewing needle was used to thread two layers of appropriate shade cotton around the iris wheel (Figure 5). The iris was positioned using the marked wax centre point and a 3 mm depth hole was made at the centre point of the sclera. The outer face and edges of the sclera prosthesis were reduced using an acrylic bur by firstly making 1 mm depth cuts and then joining the depth cuts to allow sufficient space for the iris and clear acrylic resin (Figure 6). The iris centre point was used to position the iris within the sclera and a seating ledge was created just inside the front face of the sclera prosthesis to hold its position. The pupil was positioned over the centre point of the iris and both components were fixed with cold cure acrylic resin (Vertex Dental, Netherlands) under water immersion and 3 MPa for 3 h in a water bath at 100°C.

**FIGURE 7:**
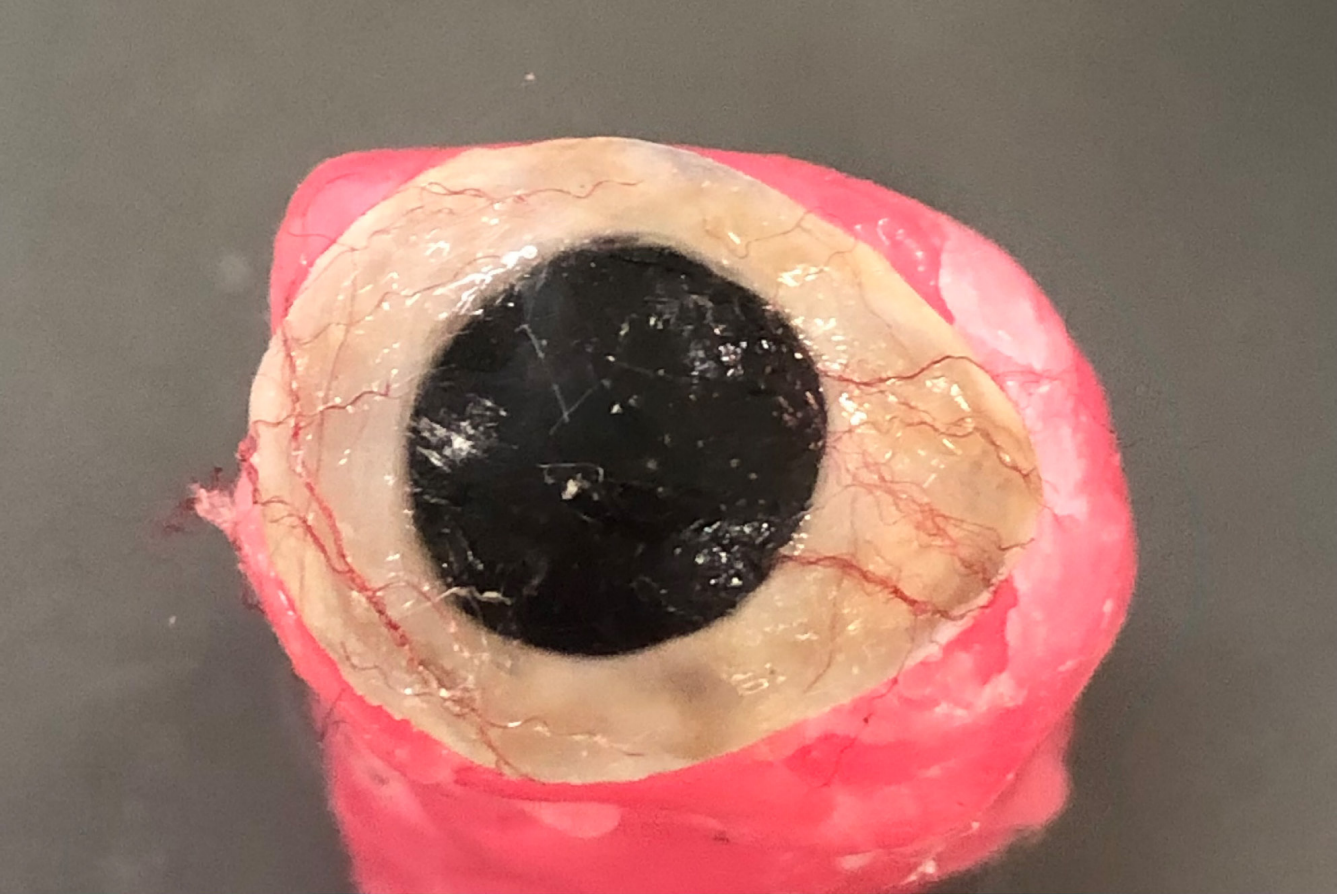
Characterization of sclera.

The sclera was characterized guided by photographs and with the patient in attendance using monomer to thin the characterizing paint. A blue layer was firstly applied to add translucency with yellow, brown or red colors added as required. Small cut threads of red acrylic wool were added to simulate blood vessels and secured with unfilled resin (Monopoly Syrup, Factor II, USA) (Figure 7). The prosthesis was positioned back in the original greenstone double flask and packed with heat cured acrylic resin and cured under 3 MPa for 3 h in a water bath at 100°C. The prosthesis was initially polished with acrylic burs then using wheel with pumice and finally with denture polishing paste.

**FIGURE 8:**
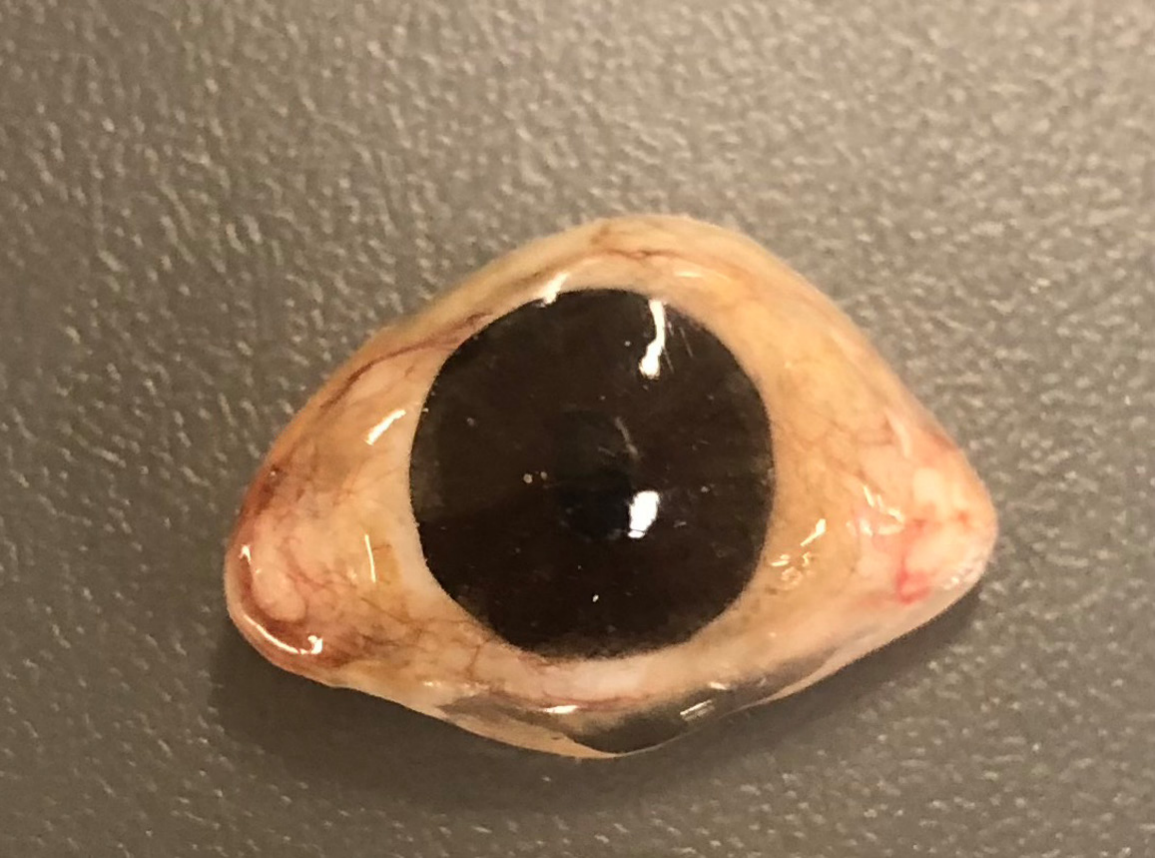
Final ocular prosthesis.

**FIGURE 9:**
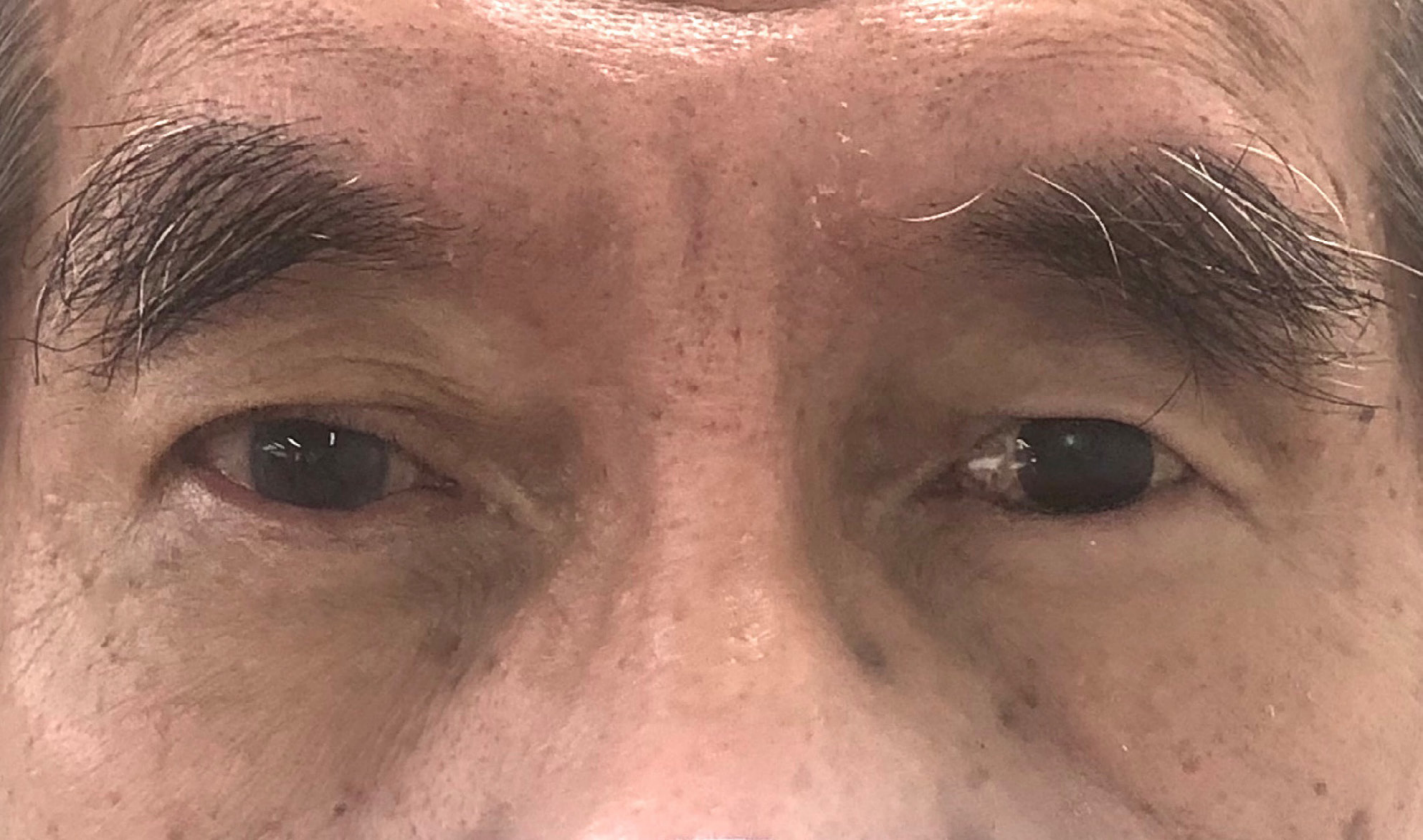
Final ocular prosthesis at insertion.

The final prosthesis was delivered to the patient and instructions for placement, removal, cleaning and storage provided (Figures 8 and 9). The prosthesis provided a satisfactory aesthetic outcome and at the one‐week review appointment the tissue bed was healthy and the patient reported high levels of satisfaction.

## 3 DISCUSSION

The most common techniques used to construct an ocular prosthesis are empirical fitting of a stock eye, modifying a stock eye through making an impression of the ocular defect and the fully customized construction technique.^12^ It is preferable to have intimate contact between the ocular prosthesis and the tissue bed to distribute even pressure, so in this regard a prefabricated prosthesis has the potential for an inferior outcome.^3^ Voids in the prefabricated prosthesis collect mucus and debris and can irritate the mucosa and act as a potential source of infection, which are minimized in the custom‐made prosthesis.^3^ The presented patient's previous ocular prostheses were predominantly prefabricated types which aligns with this philosophy.

The presented case was managed using traditional laboratory techniques some of which have similarities to denture fabrication procedures. The complexities in managing the case were highlighted by the focus on the laboratory stages of the treatment that required high time and resource inputs for a single prosthesis. The iris was constructed using our novel customized threaded iris technique which proposes more lifelike aesthetics due to the detailed construction inputs and serrated iris surface texture. The prefabricated painted ocular disc is the more traditional iris construction option with inferior optical properties and significantly shorter construction time. This case report represents the first known presentation of the threaded technique and certainly warrants further investigations.

It has been proposed that digital pathways present improved economic and time efficiencies over traditional techniques^13^ however it has not yet been possible to implement a fully digital ocular prosthesis construction pathway involving scanning. This requires ocular prosthesis construction to use either a completely traditional pathway or a combination of traditional techniques and elements of digital technology as appropriate. Digital technology has been successfully utilized in auricular prosthesis construction where the precision and accuracy of 3D ear images captured with a smartphone were similar to laser scanned stone models fabricated by traditional methods.^14^

The educational benefits and success of maxillo‐facial treatment completed in a postgraduate prosthodontics clinical residency program have been previously reported.^15^ The discipline is a niche sub‐specialty and remains a required component in postgraduate prosthodontics curricula with the potential to significantly impact the lives of a small number of patients. The most gratifying aspect of managing the presented case and providing a life‐like appearance was the patient's response to receiving the prosthesis.

## AUTHOR CONTRIBUTIONS

**James Dudley:** Conceptualization; investigation; methodology; project administration; supervision; writing – original draft; writing – review and editing. **Jane Pellew:** Conceptualization; data curation; formal analysis; investigation; methodology; writing – original draft. **Nafij Bin Jamayet:** Conceptualization; data curation; methodology; project administration; supervision; writing – original draft.

## FUNDING INFORMATION

There are no funding sources for this research.

## CONFLICT OF INTEREST STATEMENT

The authors declare no conflict of interest regarding the publication of this paper.

## ETHICS STATEMENT

Informed consent was obtained from the patient.

## CONSENT STATEMENT

Written informed consent was obtained from the patient to publish this report in accordance with the journal's patient consent policy.

## Data Availability

Data sharing is not applicable to this article as no new data were created or analyzed in this study.

## References

[1] The glossary of prosthodontic terms: Ninth edition. J Prosthet Dent. 2017;117:e1‐e105. 28418832 10.1016/j.prosdent.2016.12.001

[2] Tang RY . Role of the general dentist in maxillofacial prosthetics. J Prosthet Dent. 1976;36:416‐420. 787508 10.1016/0022-3913(76)90165-7

[3] Beumer J III , Marunick MT , Esposito SJ . Maxillofacial Rehabilitation: Prosthodontic and Surgical Management of Cancer Related, Acquired, and Congenital Defects of the Head and Neck. 3rd ed. Quintessence; 2011:61‐254.

[4] Kim J‐H , Shin S‐Y , Paek J , Lee J‐H , Kwon H‐B . Analysis of maxillofacial prosthetics at university dental hospitals in the capital region of Korea. J Adv Prosthdont. 2016;8:229‐234. 10.4047/jap.2016.8.3.229 PMC4919495 27350859

[5] Mueller S , Hohlweg‐Majert B , Buergers R . The functional and aesthetic reconstruction of midfacial and orbital defects by combining free flap transfer and craniofacial prosthesis. Clin Oral Investig. 2015;19:413‐419. 10.1007/s00784-014-1243-0 24771201

[6] Duke‐Elder WS . Textbook of Ophthalmology. Henry Kimpton; 1932:363.

[7] Jamayet N , Kirangi J , Husein A , Alam M . A comparative assessment of prosthetic outcome on enucleation and evisceration in three different etiological eye defects: a case series. Eur J Dent. 2017;11:130‐134. 28435380 10.4103/1305-7456.202636 PMC5379827

[8] Taylor TD . Clinical Maxillofacial Prosthetics. Quintessence; 2000:233‐276.

[9] Perman KI , Baylis HI . Evisceration, enucleation, and exenteration. Otolaryngol Clin North Am. 1988;21:171‐182. 3277114

[10] Wolfaardt J , Gehl G , Farmand M , Wilkes G . Indications and methods of care for aspects of extraoral osseointegration. Int J Oral Maxillofac Surg. 2003;32:124‐131. 12729770 10.1054/ijom.2002.0340

[11] Dental Board of Australia . Specialist competencies. Accessed October 4, 2022. http://www.dentalboard.gov.au/Registration/Specialist‐Registration/Specialist‐competencies.aspx

[12] Artopoulou II , Montgomery PC , Wesley PJ , Lemon JC . Digital imaging in the fabrication of ocular prostheses. J Prosthet Dent. 2006;95:327‐330. 16616132 10.1016/j.prosdent.2006.01.018

[13] Ko J , Kim SH , Baek SW , Chae MK , Yoon JS . Semi‐automated fabrication of customized ocular prosthesis with three‐dimensional printing and sublimation transfer printing technology. Sci Rep. 2019;9(1):2968. 30814585 10.1038/s41598-019-38992-y PMC6393501

[14] Farook TH , Rashid F , Jamayet NB , Abdullah JY , Dudley J , Khursheed Alam M . A virtual analysis of the precision and accuracy of 3‐dimensional ear casts generated from smartphone camera images. J Prosthet Dent. 2022;128(4):830‐836. 33642077 10.1016/j.prosdent.2020.12.041

[15] Dudley J , Mughal F , Hotinski E , Mahmud M . Prosthodontic management of maxillofacial cases: a case series. Aust Dent J. 2018;63:124‐128. 28853144 10.1111/adj.12563

